# Transfer RNA Modification Enzymes from Thermophiles and Their Modified Nucleosides in tRNA

**DOI:** 10.3390/microorganisms6040110

**Published:** 2018-10-20

**Authors:** Hiroyuki Hori, Takuya Kawamura, Takako Awai, Anna Ochi, Ryota Yamagami, Chie Tomikawa, Akira Hirata

**Affiliations:** Department of Materials Science and Biotechnology, Graduate School of Science and Engineering, Ehime University, Bunkyo 3, Matsuyama, Ehime 790-8577, Japan; t.kwmr.0115@gmail.com (T.K.); takak0v0@gmail.com (T.A.); annaoti009@yahoo.co.jp (A.O.); w844038k@yahoo.co.jp (R.Y.); tomikawa.chie.mm@ehime-u.ac.jp (C.T.); hirata.akira.mg@ehime-u.ac.jp (A.H.)

**Keywords:** archaea, methylation, pseudouridine, RNA modification, tRNA methyltransferase, tRNA modification

## Abstract

To date, numerous modified nucleosides in tRNA as well as tRNA modification enzymes have been identified not only in thermophiles but also in mesophiles. Because most modified nucleosides in tRNA from thermophiles are common to those in tRNA from mesophiles, they are considered to work essentially in steps of protein synthesis at high temperatures. At high temperatures, the structure of unmodified tRNA will be disrupted. Therefore, thermophiles must possess strategies to stabilize tRNA structures. To this end, several thermophile-specific modified nucleosides in tRNA have been identified. Other factors such as RNA-binding proteins and polyamines contribute to the stability of tRNA at high temperatures. *Thermus thermophilus*, which is an extreme-thermophilic eubacterium, can adapt its protein synthesis system in response to temperature changes via the network of modified nucleosides in tRNA and tRNA modification enzymes. Notably, tRNA modification enzymes from thermophiles are very stable. Therefore, they have been utilized for biochemical and structural studies. In the future, thermostable tRNA modification enzymes may be useful as biotechnology tools and may be utilized for medical science.

## 1. Introduction

**Figure 1:**
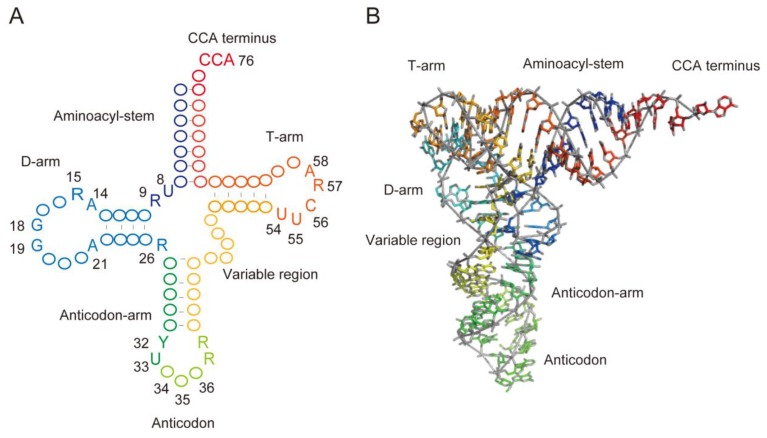
The structure of tRNA. (**A**) Representation of secondary structure of tRNA in a cloverleaf structure. This figure shows tRNA with a short variable region. Conserved nucleosides are shown with position numbers. Abbreviations: R, purine. Y, pyrimidine. (**B**) The L-shaped structure of *Saccharomyces cerevisiae* tRNA^Phe^. The colors of nucleosides correspond to those in (**A**).

Transfer RNA is an adaptor molecule required for the conversion of genetic information encoded by nucleic acids into amino acid sequences of proteins [1,2]. Figure 1A shows typically conserved nucleosides in a tRNA molecule, which is represented as a cloverleaf structure (herein, the nucleotide positions in tRNA are numbered, according to Sprinzl et al. [3]). These conserved nucleotides are important for tRNA folding and for stabilization of the L-shaped tRNA structure (Figure 1B) [4,5,6]. In addition to the standard nucleosides, numerous modified nucleosides in tRNA (for structures, see the MODOMICS and tRNAmodviz databases: http://modomics.genesilico.pl/; http://genesilico.pl/trnamodviz [7]) have been discovered in both thermophilic and mesophilic tRNAs [7,8] (see Supplementary Table S1 for abbreviations of modified nucleosides).

A comprehensive review of the modified nucleosides in tRNA from thermophiles and their positions, distribution, predicted (or confirmed) tRNA modification enzymes and structural effects (Table 1) [9,10,11,12,13,14,15,16,17,18,19,20,21,22,23,24,25,26,27,28,29,30,31,32,33,34,35,36,37,38,39,40,41,42,43,44,45,46,47,48,49,50,51,52,53,54,55,56,57,58,59,60,61,62,63,64,65,66,67,68,69,70,71,72,73,74,75,76,77,78,79,80,81,82,83,84,85,86,87,88,89,90,91,92,93,94,95,96,97,98,99,100,101,102,103,104,105,106,107,108,109,110,111,112,113,114,115,116,117,118,119,120,121,122,123,124,125,126,127,128,129,130,131,132,133,134,135,136,137,138,139,140,141,142,143,144,145,146,147,148,149,150,151,152,153,154,155,156,157,158,159,160,161,162,163,164,165,166,167,168,169,170,171,172,173,174,175,176,177,178,179,180,181,182,183,184,185,186,187,188,189,190,191,192,193,194,195,196,197,198,199,200,201,202,203,204,205,206,207,208,209,210,211,212,213,214,215,216,217,218,219,220,221,222,223,224,225,226,227,228,229,230,231,232,233,234,235,236,237,238,239,240,241,242,243,244,245,246,247,248,249,250,251,252,253,254,255,256,257,258,259,260,261,262,263,264], which suggests that the majority of modified nucleosides in tRNA from thermophiles are common to those in tRNA from mesophiles. The functions of modified nucleosides in tRNAs have been gradually elucidated by biochemical and structural studies, physicochemical measurements, and analyses of gene disruption strains. The modified nucleosides primarily function in protein synthesis (e.g., stabilization of tRNA structure [88,265,266,267], correct folding of tRNA [88,265,266,267], reinforcement, restriction, and/or alteration of codon-anticodon interaction [108,109,114,115,116,117,120,124,268,269,270], recognition by aminoacyl-tRNA synthetases [109,116,117,271], recognition by translation factors [272], and prevention of the frameshift error [122,123,157,158] among others). In short, living organisms cannot synthesize proteins correctly or efficiently without modifications in tRNA.

For some organisms, modifications in tRNA have not been confirmed, but the tRNA modification enzymes have been studied. For example, although no tRNA sequences from *Thermotoga maritima* have been reported, the properties of several tRNA modification enzymes of this hyper-thermophilic eubacterium have been documented and, thus, the nucleoside modifications are predicted. Although many of the functions and biosynthesis pathways of modified nucleosides in tRNA from thermophiles have not yet been investigated, most of them are considered to be basically common to those from mesophiles. However, thermophiles live in extreme environments (e.g., high temperature, anaerobic conditions, extreme pH, and high pressure). Therefore, it is possible that tRNA modifications observed in thermophiles may have novel functions. Furthermore, in some cases, the biosynthesis pathways of some modifications may differ between thermophiles and mesophiles. Moreover, in eukaryotes, tRNA modifications are related to higher biological processes such as cellular transport of tRNA [273,274,275,276,277,278], RNA quality control [274,279,280,281], infection [282,283,284,285,286], and the immune response [287,288,289,290]. As yet, modified nucleosides in tRNA from thermophilic eukaryotes have not been investigated, but it is possible that a relationship between modified nucleosides in tRNA and these biological phenomena may also be discovered in thermophilic eukaryotes.

In this review, we focus on the modified nucleosides and tRNA modification enzymes from thermophiles including the difficulties in sequencing the rigid and stable tRNAs from thermophiles. Since the tRNA modifications in moderate thermophiles are essentially similar to those in mesophiles, we describe them separately from extreme-thermophiles and hyper-thermophiles. We focus on the strategies for tRNA stabilization of extreme hyperthermophiles. Furthemrore, we describe the potential effects of these modifications during oxidative and other environmental stresses at high temperatures. Lastly, we describe biotechnological and therapeutic uses for tRNA modification enzymes. To avoid overlap with previous publications, we intentionally refer to representative articles and reviews of modified nucleosides in tRNA and tRNA modification enzymes from mesophiles (main text and Table 1) to aid understanding by the readers. For example, tRNA modifications in archaea including mesophiles have been extensively covered [48,87,291,292,293,294] and pseudouridine modifications and methylated nucleosides in tRNA are reviewed elsewhere [87,203,295,296]. Furthermore, the stability of nucleic acids at high temperatures has been reviewed [297]. Other useful publications are pointed out in the appropriate sections throughout the review.

## 2. Sequencing of tRNA from Thermophiles

**Figure 2:**
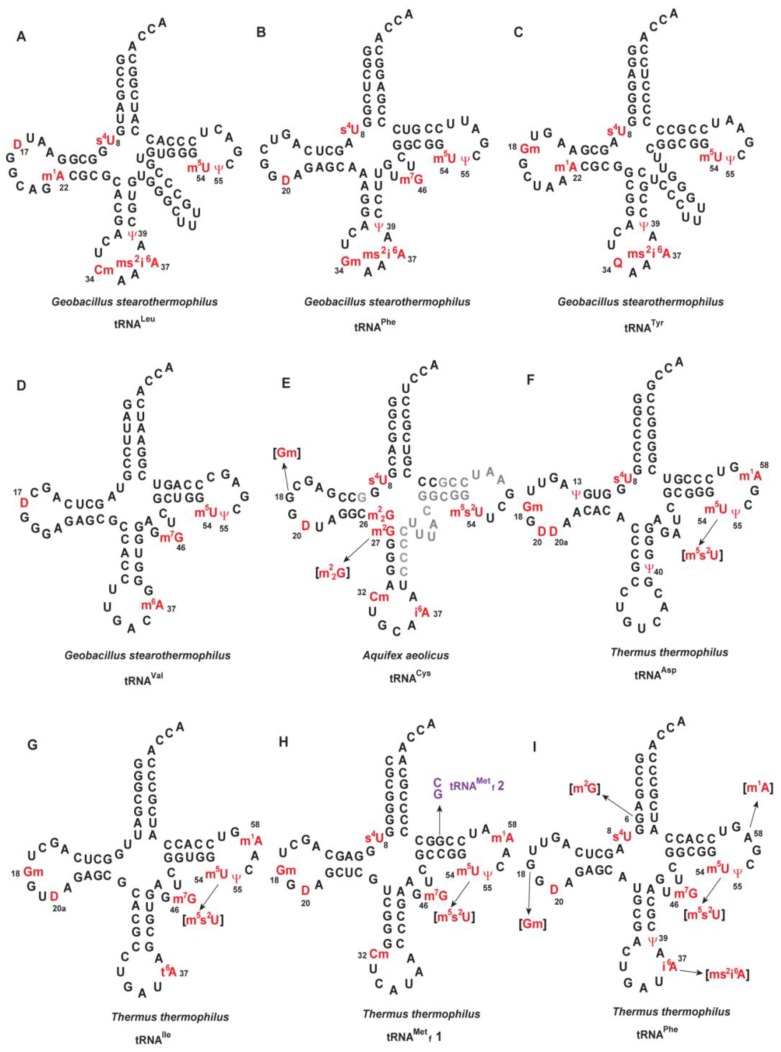

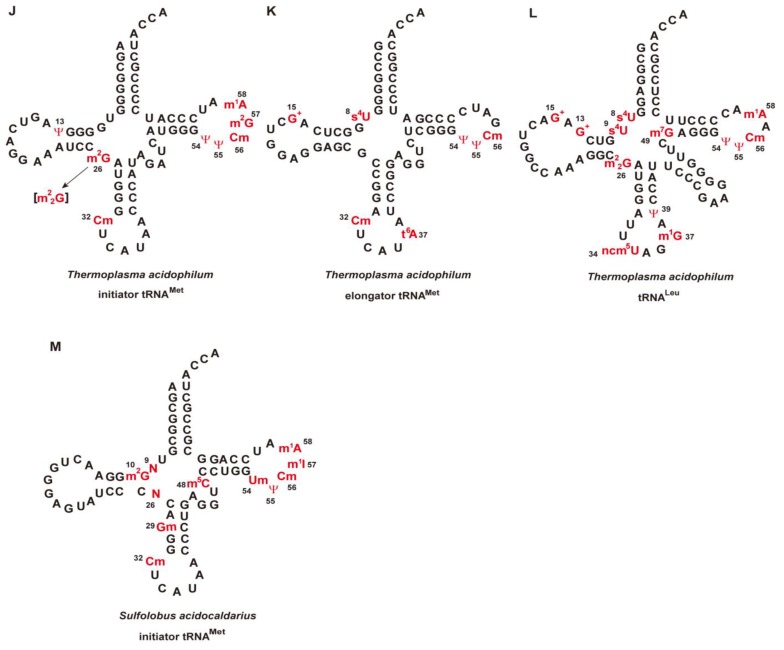
Sequences of tRNA from thermophiles. The modified nucleosides are indicated in red with their positions. Parentheses indicate that a portion of the modified nucleoside is further modified to its derivative. Abbreviations of modified nucleosides are given in Supplementary Table S1. (**A**) *Geobacillus stearothermophilus* tRNA^Leu^. (**B**) *G. stearothermophilus* tRNA^Phe^. (**C**) *G. stearothermophilus* tRNA^Tyr^. (**D**) *G. stearothermophilus* tRNA^Val^. (**E**) *Aquifex aeolicus* tRNA^Cys^. (**F**) *Thermus thermophilus* tRNA^Asp^. (**G**) *T. thermophilus* tRNA^Ile^. (**H**) *T. thermophilus* tRNA^Met^_f_1. (**I**) *T. thermophilus* tRNA^Phe^. (**J**) *Thermoplasma acidophilum* initiator tRNA^Met^. (**K**) *T. acidophilum* elongator tRNA^Met^. (**L**) *T. acidophilum* tRNA^Leu^. (**M**) *Sulfolobus acidocaldarius* initiator tRNA^Met^ In *A. aeolicus* tRNA^Cys^ (E) the nucleotides shown in gray could not be determined and cyanoethylated tRNA^Cys^ was not analyzed. Therefore, this tRNA may possess additional modifications (e.g., Ψ_39_, Ψ_55_ and m^1^A58). *Thermus thermophilus* possesses two tRNA^Met^_f_ species. The difference of tRNA^Met^_f_2 is single G-C base pair, which is indicated in purple in (H). In *S. acidocaldarius* initiator tRNA^Met^ (M), the nucleosides at positions 9 and 26 may be m^1^A_9_ and m^2^_2_Gm_26_, respectively.

The sequence of tRNA provides the most basic information of tRNA. However, as shown in Figure 2, which displays nucleotide sequences of tRNAs from thermophilic eubacteria [10,11,18,19,20,21,22,23,24,64,67] and archaea [25,36,44,252], the sequences of only 14 tRNA species have been reported from thermophiles. In addition, in the case of *Aquifex aeolicus* tRNA^Cys^, the sequence has been only partially determined [24].

In general, sequencing of tRNA from thermophiles is difficult for the following reasons. First, purification of specific tRNA from thermophiles is not easy. Currently, tRNA is purified by the solid DNA probe method [298,299,300]. In this method, the solid-phase complementary DNA probe is placed in a column and hybridized with the target tRNA and then the target tRNA is eluted from the column. Since the structures of tRNA from thermophiles are very rigid, denaturing the tRNA to allow hybridization is difficult. This problem has been solved by incorporating tetraalkyl-ammonium salt in the hybridization buffer [301]. This salt destabilizes the secondary and tertiary structures of tRNA and promotes formation of the RNA-DNA hetero-duplex. This alteration enabled us to purify *A. aeolicus* tRNA^Cys^ [24], *Thermus thermophilus* tRNA^Phe^ [11], tRNA^Met^_f_1 [248] and tRNA^Thr^ [263,302], *Thermoplasma acidophilum* initiator tRNA^Met^ [89], elongator tRNA^Met^ [89], and tRNA^Leu^ [36]. Even with the use of tetraalkyl-ammonium salt, however, the solid DNA probe method is not versatile. For example, because the difference between *T. thermophilus* tRNA^Met^_f_1 and tRNA^Met^_f_2 is only one G-C base pair in the T-stem (Figure 2H) [21], purification of tRNA^Met^_f_1 required its separation from tRNA^Met^_f_2 by BD-cellulose column chromatography before the solid DNA probe method could be applied [248].

Second, since the structure of tRNA from thermophiles is rigid, limited cleavage by formamide [303,304] is difficult. Therefore, it is difficult to apply the classical technique used for RNA sequencing to tRNA from thermophiles. Liquid-chromatography/mass-spectrometry (LC/MS) has been found to be the most reliable method to overcome this problem [305,306]. In general, LC/MS requires prior cleavage of tRNA by RNases. However, because the G-C content in the stem regions of tRNA from thermophiles is very high (Figure 2), RNA fragments with the same sequences are often generated by RNase cleavage. Therefore, use of multiple RNases and/or preparation of gene disruptant strains are required to overcome this problem.

Furthermore, given that it is not possible to distinguish uridine and pseudouridine by MS, cyanoethylation of tRNA is generally required to detect this nucleoside [307]. In the sequencing of *T. acidophilum* tRNA^Leu^ [36], we used a combination of the cyanoethylation and classical formamide method for detection of Ψ_54_ because the efficiency of cyanoethylation of Ψ_54_ was low. Thus, specific techniques are required even if an LC/MS system is available.

Third, to determine the modified nucleoside precisely, preparation of a standard compound is often required. For example, it was necessary to prepare the standard ncm^5^U nucleoside from the *Saccharomyces cerevisiae trm9* gene disruptant strain [308] to determine the anticodon modification of *T. acidophilum* tRNA^Leu^ [36]. In some cases, synthesis of a standard compound by organic chemistry may be required. Lastly, preparing cultures of thermophiles is not so easy for general biochemical researchers (e.g., under anaerobic conditions at high temperatures).

To overcome these problems, the cooperation of researchers in different fields is required. At present, the solid DNA probe method with tetraalkyl-ammonium coupled with LC/MS is the main method for sequencing tRNA from thermophiles. Therefore, it is anticipated that a large numbers of sequences of tRNA from thermophiles will be reported by using this approach in the future.

## 3. Modified Nucleosides in tRNA from Moderate Thermophiles Are Common to Those from Mesophiles

Seven tRNA sequences from moderate thermophiles (*Geobacillus stearothermophilus* and *T. acidophilum*), which live at below 75 °C, have been reported (Figure 2). Furthermore, the modified nucleosides in unfractionated tRNA from moderate thermophiles (*Methanobacterium thermoaggregans*, *Methanobacterium thermoautotrophicum*, and *Methanococcus thermolithotrophicus*) were analyzed [97,99]. These studies have shown that the modified nucleosides in tRNA from moderate thermophiles are typically common to those in tRNA from mesophiles. In summarizing the information on tRNA modifications and tRNA modification enzymes by thermophilic species [8,9,10,11,12,13,14,15,16,17,18,19,20,21,22,23,24,25,26,27,28,29,30,31,32,33,34,35,36,37,38,39,40,41,42,43,44,45,46,47,48,49,50,51,52,53,54,55,56,57,58,59,60,61,62,63,64,65,66,67,68,69,70,71,72,73,74,75,76,77,78,79,80,81,82,83,84,85,86,87,88,89,90,91,92,93,94,95,96,97,98,99,100,101,102,103,104,105,106,107,108,109,110,111,112,113,114,115,116,117,118,119,120,121,122,123,124,125,126,127,128,129,130,131,132,133,134,135,136,137,138,139,140,141,142,143,144,145,146,147,148,149,150,151,152,153,154,155,156,157,158,159,160,161,162,163,164,165,166,167,168,169,170,171,172,173,174,175,176,177,178,179,180,181,182,183,184,185,186,187,188,189,190,191,192,193,194,195,196,197,198,199,200,201,202,203,204,205,206,207,208,209,210,211,212,213,214,215,216,217,218,219,220,221,222,223,224,225,226,227,228,229,230,231,232,233,234,235,236,237,238,239,240,241,242,243,244,245,246,247,248,249,250,251,252,253,254,255,256,257,258,259,260,261,262,263,264,265,266,267,268,269,270,271,272,273,274,275,276,277,278,279,280,281,282,283,284,285,286,287,288,289,290,291,292,293,294,295,296,297,298,299,300,301,302,303,304,305,306,307,308,309,310,311,312,313,314,315,316,317,318,319,320,321,322,323,324,325,326,327,328,329,330] (Table 2), we have separately considered moderate thermophiles, extreme-thermophiles, and hyper-thermophiles. However, there are some differences between moderate thermophiles and mesophiles. For example, the degree of 2’-*O*-methylation in tRNA from *G. stearothermophilus* is increased at high temperatures [309]. Furthermore, several modifications (Gm_18_, D modifications, and Gm_34_) in tRNA from *G. stearothermophilus* cannot be explained by the enzymatic activities of the already-known tRNA modification enzymes, which is described in Table 2. Moreover, *T. acidophilum* possesses several distinct tRNA modifications such as G^+^_13_ and m^7^G_49_ (Table 2) [36]. Although these differences are present, thermophile-specific modified nucleosides have not been found in tRNA from moderate thermophiles, which suggests that living organisms can survive at 75 °C via the tRNA modifications in mesophiles.

## 4. Strategies of tRNA Stabilization by Modified Nucleosides in Extreme-Thermophiles and Hyper-Thermophiles

**Figure 3:**
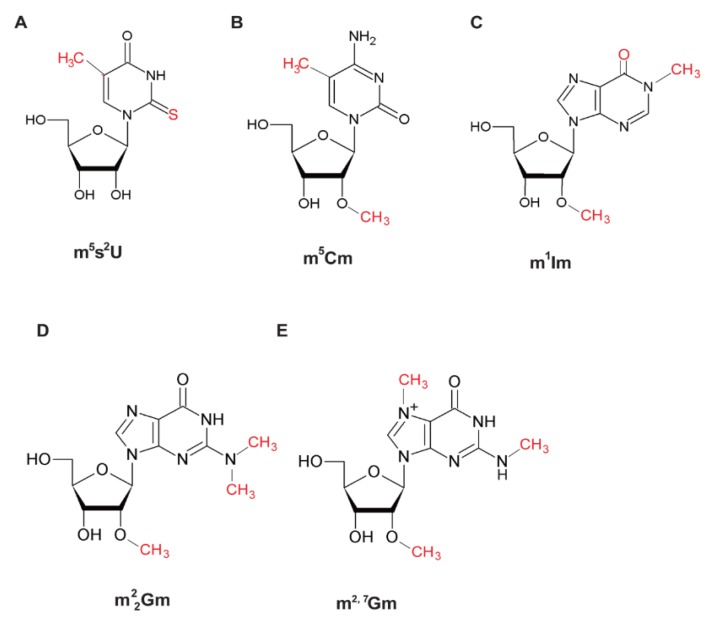
Thermophile-specific modified nucleosides in tRNA. Abbreviations of modified nucleosides are given in Supplementary Table S1. (**A**) m^5^s^2^U. (**B**) m^5^Cm. (**C**) m^1^Im. (**D**) m^2^_2_Gm. (**E**) m^2, 7^Gm. The modifications are indicated in red.

In general, the G-C content in the stem regions of tRNA from thermophiles is very high (Figure 2). However, the stability of tRNA from thermophiles cannot be explained only by the increase in G-C content in the stem region. For example, although the melting temperature of *T. thermophilus* tRNA^Phe^ transcript is 76 °C, that of the native tRNA^Phe^ is 84.5 °C [11]. Thus, modified nucleosides are essentially required for stabilization of tRNA at high temperatures. Modified nucleosides in tRNA from thermophiles have been studied mainly from the view point of tRNA stabilization. So far, only a few modified nucleosides specific to thermophiles have been found (Figure 3). These thermophile-specific modified nucleosides seem to stabilize the tRNA structures at high temperatures. As described below, extreme-thermophiles and hyper-thermophiles possess two strategies of tRNA stabilization by modified nucleosides. One is based on thermophile-specific modification such as m^5^s^2^U_54_ (Figure 3A) and the other is based on 2′-*O*-methylations at multiple positions in tRNA (Figure 3B–E). Recently, the unknown modified nucleoside at position 26 in *Sulfolobus acidocaldarius* tRNA^Met^ (Figure 2M) was described as m^2^_2_Gm [96]. On the whole, however, the modification site(s), modified tRNA species, and biosynthesis pathways of most thermophile-specific modified nucleosides are unknown. Moreover, these nucleosides may have additional functions at high temperatures beyond their structural effect.

### 4.1. m^5^s^2^U_54_ Is a Typical Thermophile-Specific Modified Nucleoside in tRNA

The m^5^s^2^U_54_ modification was originally found in tRNA from *T. thermophilus* [331]. Subsequently, this modified nucleoside was found in tRNA from *A. aeolicus*, *T. maritima*, *Pyrococcus abyssi, Pyorococcus horikoshii*, and *T. kodakarensis* (Table 2) but not from mesophiles. The m^5^s^2^U_54_ modification forms a reverse Hoogsteen base-pair with A_58_ (or m^1^A_58_) in tRNA and stabilizes the tRNA structure by stacking with the G_51_–C_61_ base-pair [220]. Because the 2-thio-modification at position 54 increases the melting temperature of tRNA by more than 3 °C [22,218,220], the m^5^s^2^U_54_ modification contributes to stabilization of the tRNA structure. The degree of m^5^s^2^U_54_ modification increases with an increasing temperature [22,67,220,229]. At 80 °C, the extent of m^5^s^2^U_54_ modification in tRNA is almost 100% [22,67,220,229]. The melting temperature of tRNA mixture is maintained above 85 °C due to the presence of m^5^s^2^U_54_ modification [229] and *T. thermophilus* can grow at 50 to 83 °C. Thus, living organisms can survive at 80 °C due to the presence of m^5^s^2^U_54_ modification in tRNA.

### 4.2. The Network Between Modified Nucleosides in tRNA and tRNA Modification Enzymes in T. thermophilus Adapts Protein Synthesis at Low and High Temperatures

Under natural conditions, the temperature of hot spring water fluctuates for several reasons including an influx of river water, snowfall, and an eruption of hot water. In accordance with these temperature changes, *T. thermophilus* can synthesize proteins efficiently at a wide range of temperatures (50 to 83 °C) by regulating the flexibility (rigidity) of its tRNA [220]. At high temperatures (above 75 °C), three modified nucleosides in tRNA, m^5^s^2^U_54_ [230], m^1^A_58_ [260], and m^7^G_46_ [11] are essential for survival of *T. thermophilus*. The m^1^A_58_ modification is one of the positive determinants for the two-thiolation system of m^5^s^2^U_54_. Thus, a *T. thermophilus* disruptant strain of the *trmI* gene encoding the tRNA m^1^A_58_ methyltransferase cannot grow at 80 °C [229,260]. The presence of m^7^G_46_ modification in tRNA increases the speed of tRNA modification enzymes such as TrmH for Gm_18_, TrmD for m^1^G_37_, and TrmI for m^1^A_58_ [11]. The m^1^A_58_ modification further increases the rate of sulfur-transfer to m^5^U_54_ by the 2-thiolation system and the introduced modified nucleosides coordinately stabilize the tRNA structure. Thus, the m^7^G_46_ modification produced by TrmB is a key factor in the network between modified nucleosides in tRNA and tRNA modification enzymes of *T. thermophilus* at high temperatures. In the *trmB*-gene disruptant starin, tRNA^Phe^ and tRNA^Ile^ were found to be degraded by a temperature shift from 70 °C to 80 °C and heat-shock proteins were not synthesized efficiently [11].

At low temperatures (below 55 °C), in contrast, the Ψ_55_ modification produced by TruB is essential for the survival of *T. thermophilus* [248]. The presence of Ψ_55_ stabilizes both the local structure of the T-arm and the interaction of the T-arm with the D-arm in tRNA. The local rigidity in tRNA caused by Ψ_55_ slows down the speeds of introducing modified nucleosides around Ψ_55_ (Gm_18_, m^5^s^2^U_54_ and m^1^A_58_), which maintains the flexibility of tRNA at low temperatures. The presence of m^5^U_54_ modification by TrmFO supports this effect of Ψ_55_ [225].

It should be mentioned that D modifications are thought to bring flexibility to tRNA because D does not stack with other bases and brings about the C2′-endo form of ribose [332]. However, a *T. thermophilus* disruptant strain of the *dusA* gene encoding tRNA D_20_/D_20a_ synthase did not show growth retardation at 50, 60, 70, or 80 °C, and abnormal modifications were not observed in tRNA from this strain [85]. Therefore, the function of D_20_ and D_20a_ modifications is unknown. Since DusA recognizes the interaction of T-arms and D-arms in tRNA [84], the stabilization of the L-shaped tRNA structure by other modified nucleosides is required for the efficient introduction of D_20_ and D_20a_ at high temperatures [85]. Thus, D_20_ and D_20a_ are relatively late modifications in *T. thermophilus* tRNA.

Although the above network is a temperature adaptation system of *T. thermophilus,* it regulates the order in which modified nucleosides are introduced into tRNA. Similar networks have been found in mesophiles [333]. In *Escherichia coli*, for example, the 2′-*O*-methylation at position 34 by TrmL requires an i^6^A_37_ modification [334]. However, the network in *T. thermophilus* is distinct because it regulates the structure of a three-dimensional core and many modifications in tRNA are related. One of the advantages of this system is that protein synthesis is not required. The response of the system is very rapid. It is possible that thermophilic archaea possess a similar network between modified nucleosides in tRNA and tRNA modification enzymes because some of them can also grow at a wide range of temperatures.

### 4.3. Stabilization of tRNA Structure by 2′-O-Methylation

Because 2′-*O*-methylation shifts the equilibrium of ribose puckering to the C3′-endo form and enhances the hydrophobic interaction, this modification, when carried out at multiple positions, brings rigidity of tRNA. Furthermore, 2’-*O*-methylations prevents hydrolysis of phophodiester-bonds in tRNA at high temperatures. Therefore, 2’-*O*-methylations may prolong the half-lives of tRNA. Notably, there is a living organism in which tRNA is stabilized without m^5^s^2^U_54_ modification. A hyper-thermophilic archaeon, *Pyrodictium occultum* can grow at 105 °C, and various 2′-*O*-methylted nucleosides such as Ψm, m^1^Im, and m^2^_2_Gm are present in its tRNA, but s^2^U and m^5^s^2^U are not observed [97,98]. Notably, although the melting temperature of the *P. occultum* tRNA^Met^ transcript is 80 °C, that of the native tRNA^Met^ is more than 100 °C [323]. Thus, the melting temperature of *P. occultum* tRNA is increased by more than 20 °C through a combination of numerous 2′-*O*-methylated nucleosides.

*Methanopyrus kandleri* can grow at more than 110 °C and tRNAs from this archaeon contain many unique modifications such as U_8_ (the product of C_8_ to U_8_ editing) [16], ac^6^A, m^2, 7^Gm, and methyl-hn^6^A [100]. Furthermore, *M. kandleri* possesses 132 species of C/D-box guide RNAs [17], which suggests that RNAs are highly methylated by the L7Ae, Nop5, aFib, and C/D-box guide RNA system. In the case of *M. kandleri*, therefore, tRNA seems to be stabilized by unique modifications and 2′-*O*-methylations.

These observations suggest that living organisms can survive at more than 100 °C by a combination of 2′-*O*-methylations and other thermophile-specific tRNA modifications.

### 4.4. Other tRNA Stabilization Factors

RNA binding proteins, polyamines, magnesium ions, and potassium ions are all able to stabilize tRNA in thermophiles. For example, transfer RNA-binding protein 111 (Trbp111) is an RNA-binding protein that is observed only in *A. aeolicus* [335,336,337]. *A. aeolicus* can grow at 94 °C and modified nucleosides in tRNA of this hyperthermophilic eubacterium are not so different from those in tRNA from *T. thermophilus*, which grows at temperatures below 83 °C. Therefore, Trbp111 may provide more than 10 °C of tRNA stabilization in *A. aeolicus*. The docking model of Trbp111 and tRNA suggests that Trbp111 stabilizes the three-dimensional core of tRNA [336]. Archease is another tRNA-binding protein that can change the methylation site of *P. abyssi* Trm4 [209]. Furthermore, archease promotes the ligation of tRNA exons during tRNA splicing [338,339]. Therefore, it has the potential to stabilize the tRNA structure at high temperatures.

Many tRNA-binding proteins and RNA chaperone proteins have been identified in eukaryotic cells [340,341]. Although these types of protein are unknown in thermophilic eukaryotes, some of them may stabilize the tRNA structure (or help correct folding of tRNA) at high temperatures. Recently, it was revealed that *E. coli* TruB (tRNA Ψ_55_ synthase [243]) possesses an RNA chaperone activity [342,343]. In the case of *T. thermophilus*, although the Ψ_55_ modification is required for survival at low temperatures (below 55 °C), the *truB* gene disruptant strain shows abnormal growth at 80 °C [248]. Therefore, the RNA chaperone effect of TruB may also be expressed at high temperatures in *T. thermophilus*. Furthermore, these observations suggest that other tRNA modification enzymes have the potential to work as RNA chaperones.

In general, polyamines have the potential to interact with nucleic acids and phospholipids because they possess multiple positive charges and hydrophobic areas. There are several studies on the interaction between tRNA and polyamines [344,345,346,347]. Thermophiles produce unique polyamines including long and branched polyamines [348,349,350,351]. Therefore, polyamines probably contribute to stabilize the tRNA structure at high temperatures. Furthermore, in vitro studies have shown that thermophile-specific long and branched polyamines affect the activities of several tRNA modification enzymes [81,352]. For example, TrmH from *T. thermophilus* methylates tRNA transcript at 80 °C only in the presence of long or branched polyamines [81]. Moreover, the long and branched polyamines are required for the maintenance of several tRNAs and the 70S ribosome and are essential for the survival of *T. thermophilus* at high temperatures [353].

Lastly, magnesium ions have been shown to be a tRNA stabilization factor [6,88,354] and are very important when considering the structural effects of several modified nucleosides in tRNA [58,88,354,355,356]. However, the precise concentration of magnesium ions in thermophile cells is unknown. It may differ depending on the growth environments. Potassium ions also function as RNA stabilization factor [88]. Notably, the interacellular concentration of some hyperthermophilic archaea (*M. fervidus* and *P. furiosus*) is much higher (700–900 mM) than that of mesophilic archaea [357]. In the case of *Methanothermus sociabilis*, the interacellular potassium concentration reaches 1060 mM [357]. These high concentrations of potassium ions may have effects on the stability of tRNA and the activities of tRNA modification enzymes.

## 5. tRNA Modifications and Environmental Stresses at High Temperatures

Recent studies have revealed that the modifications in tRNA are stress-resistance and/or stress-response factors [102,358,359,360,361]. Furthermore, a high temperature itself can be a stress factor for living organisms because some modified nucleosides (D and m^7^G) are liable at high temepratures [297].

### 5.1. Oxidative Stress

Many thermophiles can grow under aerobic conditions. For example, *Aerophyrum pernix* can grow at 100 °C under aerobic conditions. Under such conditions at high temperatures, living organisms seem to be exposed to heavy oxidative stress, which is a typical environmental stress. The amount of antioxidant enzymes such as superoxide-dismutase, catalase, and peroxidase in *Thermus filiformis*, which is an extreme-thermophilic eubacterium, increases at high temperatures [362].

Among tRNA modification enzymes, both Fe-S cluster proteins [34,130,134,142,150,173,196,236,363] for sulfur-transfer, reduction of base and/or radical S-adenosyl-l-methionine (SAM) reaction, and enzymes with catalytic cysteine residues [141,210,364,365,366], seem to be easily changed under oxidative stress. In some cases, the substrate (e.g., electron donors and folate derivatives [126,221,227,367]) may be unstable under aerobic conditions at high temperatures. Similarly, several modified nucleosides such as D and s^4^U may be labile under oxidative stress at high temperatures. Therefore, aerobic thermophiles need to protect their cellular components from oxidative stress and their tRNA modifications may respond to such stress as in mesophiles. Overall, however, the relationship between oxidative stress and tRNA modifications in thermopiles is unclear. In addition, tRNA modification systems in some thermophiles may utilize aerobic conditions at high temperatures. For example, *A. aeolicus* grows under microaerophilic conditions at high temperatures (80–94 °C) and the dimer structure of *A. aeolicus* TrmD is stabilized by inter-subunit disulfide bonds [165].

### 5.2. Other Environmental Stresses

Thermophiles often live in severe environments such as extreme pH and high pressure in addition to high temperatures. These environmental stresses may give rise to the diversity of tRNA modifications. At present, however, there are no data to support this viewpoint.

UV-stress is one such environmental stress and the s^4^U modification in tRNA is a known UV-stress-resistance factor for *E. coli* [368] and *Salmonella typhimurium* [27]. Thus, the s^4^U modification in tRNA is likely to work similarly to a UV-resistant factor in thermophiles. Interestingly, the genomes of *Archaeoglobus fulgidus* and *Methanocaldococcus janaschii,* which were isolated from the oil mines under the sea and deep sea, respectively, contain a *thiI* genes [369] (AF_RS04455 and MJ_RS04985, respectively) encoding tRNA s^4^U_8_ synthetase. Since sunlight does not reach the environments in which these thermophilic archaea live, the s^4^U modification and/or ThiI may have an additional function (e.g., sulfur-metabolism) in these archaea. Furthermore, it was recently reported that the melting temperature of tRNA from an *E. coli thiI*-gene disruptant strain was decreased relative to the wild-type strain [33]. Therefore, the s^4^U_8_ modification may contribute to stabilize tRNA structure. Furthermore, UV-stress may have an effect on other tRNA modifications via the cross-linking of s^4^U in tRNA. For example, the methylation speed of *T. thermophilus* TrmH is decreased when the substrate tRNA is cross-linked [30].

Lastly, the availability of nutrient-factors may have an effect on tRNA modifications in thermophiles. To test this idea, the extent of modifications in tRNA from *T. thermophilus* cells cultured in a nutrient-poor condition was investigated [227]. Contrary to expectation, the extent of the modification of all methylated nucleosides analyzed was normal, which demonstrates that the limited nutrients were preferentially consumed in the tRNA modification systems [227]. Thus, the findings indicated the importance of tRNA modifications for the survival of *T. thermophilus*.

## 6. Utilization of tRNA Modification Enzymes from Thermophiles

Given that proteins from thermophiles are heat-resistant and very stable, numerous tRNA modification enzymes have been used in biochemical and structural studies (Table 1 and Table 2). In particular, crystal structural studies of thermostable enzymes provided significant information on catalytic mechanisms and RNA-protein interactions. Studies on the crystal structures of tRNA modification enzymes from thermophiles are summarized in Supplementary Table S2. It is anticipated that thermostable proteins will continue to contribute structural studies in the future. Thermostable tRNA modification enzymes can be a tool for molecular and cell biology. For example, *A. fulgidus* TiaS with agmatine analogues has been used for site-specific RNA-labeling in mammalian cells [315]. In addition, thermostable tRNA modification enzymes may be used for healthcare. For example, Gm_18_ modification in tRNA does not stimulate the Toll-like receptor 7 [287,288] and tRNA with Gm_18_ alleviates inflammation [288]. Since TrmH from *T. thermophilus* can methylate all tRNA species [72] and is very stable, it may be useful for preparing tRNAs with Gm_18_ modifications for tRNA therapy.

## 7. Perspective

Given that the temperature of ancient Earth was very high relative to that of present-day Earth, thermophiles may be remnants of ancient living organisms. Therefore, studies on tRNA modification enzymes and modified nucleosides in tRNA from thermophiles will contribute to the considerations of the evolutionary pathways of living organisms. Furthermore, such studies will continue to shed light on the variety and environmental adaptations of living organisms. Moreover, as outlined above, the thermostable enzymes may be useful as biotechnological and medical tools and may contribute toward the production of valuable materials.

## Figures and Tables

**Table 1:** Modified nucleosides in tRNA from thermophiles.

Modified Nucleoside and Position	Distrib.	Modification Enzyme	Predicted Functions and Additional Information	References
Am_6_	A	Unknown	Stabilization of aminoacyl-stem Enzymatic activity for Am_6_ formation has been detected in the cell extract of *Pyrococcus furiosus*	[9]
m^2^G_6_	B/A	TrmN/Trm14	Stabilization of aminoacyl-stem	[10,11,12,13,14,15]
U_8_	A	CDAT8	Increasing G-C content in tRNA genes In *Methanopyrus kandleri*, U_8_ in several tRNA is produced from C_8_ by the deamination [16] In *Methanopyrus kandleri*, numerous nucleosides in RNA may be 2-*O*-methylated (see main text) [17]	[16,17]
s^4^U_8_	B/A	ThiI + IscS/ThiI	UV resistance in *E. coli* and *Salmonella typhimurium* (see main text) Stabilization of D-arm structure in *E. coli* (see main text)	[10,11,18,19,20,21,22,23,24,25,26,27,28,29,30,31,32,33,34,35]
s^4^U_8_ and s^4^U_9_	A	ThiI + α?	UV resistance Stabilization of D-arm structure (see main text) Sulfur-containing modifications in tRNA are reviewed in Reference [35].	[36]
m^1^A_9_	A	Archaeal Trm10	Stabilization of the D-arm structure Prevention of formation of a Watson–Crick base pair Correct folding of the D-arm region	[37,38]
m^1^G_9_ and m^1^A_9_	A	archaeal Trm10	Stabilization of D-arm structure Prevention of formation of a Watson–Crick base pair Correct folding of D-arm region *Thermococcus kodakarensis* Trm10 forms m^1^G_9_ and m^1^A_9_, whereas *Sulfolobus acidocaldarius* Trm10 forms only m^1^A_9_	[37,39]
(m^2^G_10_ and) m^2^_2_G_10_	A	archaeal Trm11 (Trm-G_10_; Trm-m^2^_2_G_10_ enzyme)	Prevention of formation of a Watson-Crick base pair Correct folding of tRNA in *Pyroccocus furiosus* Correct folding of the D-arm region	[40,41,42,43]
Ψ_13_	B/A	TruD/TruD or archaeal Pus7	Stabilization of D-stem structure Archaeal Pus7 generally catalyzes formation of Ψ35 in tRNA^Tyr^, but *Sulfolobus solfaraticus* Pus7 has weak Ψ13 formation activity [46]	[23,44,45,46]
G^+^_13_	A	ArcTGT + ArcS?	Stabilization of the D-arm structure *Thermoplasma acidophilum* tRNA^Leu^ exceptionally possesses a G^+^_13_ modification and *T. acidophilum* ArcTGT acts on positions 13 and 15 in this tRNA [47]	[36,47]
G^+^_15_	A	ArcTGT + ArcS or QueF-like protein	Stabilization of interaction between the D-arm and the variable region Several archaea possess a split-type ArcTGT [60,61] Several species in Crenarchaeota possess a QueF-like protein instead of ArcS [60,62,63] G^+^ is not found in nucleosides from a *Stetteria hydrogenophila* tRNA mixture [56]	[25,36,47,48,49,50,51,52,53,54,55,56,57,58,59,60,61,62,63]
D_17_	B	Dus family protein?	Maintenance of D-loop flexibility D_17_ and D_20_ modifications have been reported in *Geobacillus stearothermophilus* tRNA. However, D_17_ and D_20_ are formed by DusB and DusA, respectively, in *Escherichia coli* [65,66] and the *G. stearothermophilus* genome possesses only one *dus*-like gene. This is also observed in *Bacillus subtilis*, which is a mesophilic eubacterium.	[18,19,64,65,66]
Gm_18_	B	TrmH	Stabilization of the D-arm and the T-arm interaction. TrmH from thermophiles possess relative broad substrate tRNA specificities as compared with TrmH from *E. coli*. The substrate tRNA specificities of TrmH enzymes differ among thermophiles. TrmH from *Thermus thermophilus* can methylate all tRNA species.	[10,11,20,21,23,24,30,67,68,69,70,71,72,73,74,75,76,77,78,79,80,81]
D_20_	B	Dus family protein	Stabilization of local structure of D-loop in *E. coli*? In *A. aeolicus*, the nucleosides at positions 20 and 20a in tRNA^Cys^ are D_20_ and U_20a_, respectively. Therefore, Dus from *A. aeolicus* may act only on U_20_ in tRNA.	[24,33,65,66,82]
D_20_ and D_20a_	B	DusA	Stabilization of local structure of the D-loop The melting temperature of a tRNA mixture from the *E. coli dusA* gene disruptant strain is lower than that from the wild-type strain [33]. Therefore, D_20_ and D_20a_ modifications may contribute to stabilize local structure of the D-loop. *Thermus thermophilus* Dus was recently confirmed as a member of the DusA family [65,66,84,85].	[21,22,23,33,65,66,67,83,84,85]
m^1^A_22_	B	TrmK	Prevention of formation of a Watson–Crick base pair	[18,20,86]
Ψ_22_	A	Unknown	The Ψ_13_-Ψ_22_ base pair may stabilize D-arm structure [88]	[87,88]
m^2^G_26_ and m^2^_2_G_26_	A	Trm1	Stabilization of three-dimensional core structure Correct folding of tRNA Recently, it has been reported that m^2^_2_G_26_ modification is required for correct folding of precursor tRNA^Ser^ from *Schizosaccharomyces pombe* [94]. Therefore, a similar phenomenon may take place in thermophiles.	[9,25,44,89,90,91,92,93,94]
m^2^G_26_, m^2^_2_G_26_, m^2^G_27_ and m^2^_2_G_27_	B	Trm1	Stabilization of three-dimensional core structure in *A. aeolicus*. In the case of m^2^G_27_ and m^2^_2_G_27_, stabilization of aminoacyl-stem	[24,95]
m^2^_2_Gm_26_	A	Trm1 + unknown MT	Stabilization of three-dimensional core structure The presence of m^2^_2_Gm has been confirmed in nucleosides of a tRNA mixture from several thermophilic archaea [56,97,98,99,100]. Although the nucleoside at position 26 in *S. acidocaldarius* tRNA^Met^_i_ was originally reported as an unidentified G modification [44], it was recently described as m^2^_2_Gm [96]. The MT for 2’-*O*-methylation is unknown.	[44,96]
Cm_32_	A	archaeal TrmJ	Stabilization of anticodon-loop	[96]
Cm_32_ and Nm_32_	B	TrmJ	Stabilization of anticodon-loop TrmJ from *E. coli* does not recognize the base at position 32 [96,102]. Um_32_ and Am_32_ have not been reported in tRNAs from thermophilic eubacteria.	[96,101,102]
I_34_	B	TadA	Alteration of codon–anticodon interaction A-to-I editing in tRNA is reviewed in Reference [107]	[103,104,105,106,107]
k^2^C_34_	B	TilS	Alteration of codon–anticodon interaction (*E. coli* and *B. subtilis*) Change of recognition by aminoacyl-tRNA synthetase (*E. coli* and *B. subtilis*) Decoding of AUA codons by k^2^C_34_ and agm^2^C_34_ modifications is reviewed in References [114,115].	[108,109,110,111,112,113]
agm^2^C_34_	A	TiaS	Alteration of codon–anticodon interaction (*Arhaeoglobus fulgidus* and *Haloarcula marismourtui*) Change of recognition by aminoacyl-tRNA synthetase (*Arhaeoglobus fulgidus* and *Haloarcula marismourtui*) Decoding of AUA codons by k^2^C_34_ and agm^2^C_34_ modifications is reviewed in References [114,115].	[114,115,116,117,118,119,120]
xm^5^U_34_ derivatives	B/A	MnmE + MnmG + MnmC (for mnm^5^U_34_ in eubacteria)/Elp3? + α (for cm^5^U_34_ in archaea) IscS + TusA + TusBCD + TusE + mnmA (for 2-thiolation in *E. coli*) or YrvO + mnmA (for 2-thiolation in *B. subtilis*) SAMP2 + UbaA + NcsA (for 2-thiolation in *M. maripuludis*)	Reinforcement of codon–anticodon interaction (*E. coli* and other mesophiles) Restriction of wobble base pairing (*E. coli* and other mesophiles) Prevention of frameshift errors (*E. coli* and other mesophiles) Biosynthesis pathways of xm^5^U_34_ derivatives are not completely clarified. Although the information on xm^5^U_34_ derivatives in tRNA from thermophiles is limited, the biosynthesis pathways may be common with those from mesophiles. For the functions and biosynthesis pathways for xm^5^U_34_ derivatives, see References [121,122,123,124,125,126,127,128,129,130,131,132,136,137,138,139,142]. For the thiolation of xm^5^s^2^U34 derivatives, see References [35,133,134,135]. *Aquifex aeolicus* exceptionally possesses a DUF752 protein, which is an MT for the xm^5^U_34_ modifications without an oxidase domain [136]. A mnm^5^U nucleoside has been found in modified nucleosides from unfractionated tRNA in several methane archaea [56]. *Thermoplasma acidophilum* tRNA^Leu^ possesses ncm^5^U_34_ [36]. Some thermophiles in Euryarchaea may have a cnm^5^U_34_ modification in tRNA [137]. The cm^5^U_34_ formation activity of Elp3 from *Methanocaldococcus infernus* has been reported [142]. Several related proteins for synthesis of xm^5^U_34_ derivatives from thermophiles have been used for structural studies [136,138,139,140,141].	[34,35,36,56,121,122,123,124,125,126,127,128,129,130,131,132,133,134,135,136,137,138,139,140,141,142]
Cm_34_ and cmnm^5^Um_34_	B	TrmL	Reinforcement of codon–anticodon interaction (*E. coli*)	[18,143,144]
Gm_34_	B	Unknown	Reinforcement of codon–anticodon interaction (*G. stearothermophilus*)	[19]
Q_34_ derivatives	B	Tgt + QueA + QueG	Reinforcement of codon–anticodon interaction (*E. coli*) Prevention of frame-shift error (*E. coli*) Biosynthesis pathways and functions of Q derivatives are reviewed in References [152,153]. A crystal structure of QueA from *T. maritima* has been reported [151].	[20,122,145,146,147,148,149,150,151,152,153]
Cm_34_ and Um_39_ (or Cm_39_)	A	L7Ae + Nop5 + archaeal fibrillarin + Box C/D guide RNA (intron)	Reinforcement of codon–anticodon interaction Reinforcement of anticodon-arm In several archaea, an intron in precursor tRNA^Trp^ functions as a Box C/D guide RNA.	[9,154,155]
Ψ35	A	aPus7 and H/ACA guide RNA system	Reinforcement of codon–anticodon interaction	[46]
m^1^G_37_	B/A	TrmD/Trm5	Prevention of frame-shift error (*E. coli* and other mesophiles) Recognition by aminoacyl-tRNA synthetase (*Saccharomyces cerevisiae*)	[36,156,157,158,159,160,161,162,163,164,165,166,167,168,169,170,171]
wyosine_37_ derivatives	A	Trm5 + Taw1 + Taw2 + Taw3	Reinforcement of codon–anticodon interaction Prevention of the frame-shift error In several archaea, m^1^G_37_ in tRNA^Phe^ is further modified to wyosine derivatives. For the biogenesis pathway of wyosine derivatives, see References [181,182,183].	[172,173,174,175,176,177,178,179,180,181,182,183]
t^6^A_37_ derivatives	B/A	TsaB, TsaC (TsaC2), TsaD and TsaE/KEOPS complex: Kae1, Bud32, Cgi121 and Pcc1 + Sua5	Reinforcement of codon–anticodon interaction Prevention of frame-shift error Recognition by aminoacyl-tRNA synthetases The biogenesis pathway for t^6^A derivatives is reviewed in Reference [191]	[68,184,185,186,187,188,189,190,191]
i^6^A_37_ derivatives	B	MiaA + MiaB	Prevention of frame-shift error Reinforcement of codon–anticodon interaction Recognition by aminoacyl-tRNA synthetases i^6^A derivatives are reviewed in Reference [197]	[10,11,18,19,20,24,192,193,194,195,196,197]
m^6^A_37_	B	YfiC (TrmG?)		[64,198]
Ψ_38_, Ψ_39_ and Ψ_40_	B/A	TruA/Pus3	Prevention of frame-shift error (*E. coli*) Reinforcement of anticodon-arm	[10,11,18,19,20,23,87,199,200,201,202,203]
m^7^G_46_	B	TrmB	Stabilization of three-dimensional core In the case of *T. thermophilus*, m^7^G_46_ modification functions a key factor in a network between modified nucleosides in tRNA and tRNA modification enzymes (see main text) [11]	[10,11,19,67,204,205,206,207,208]
m^5^C_48_ and m^5^C_49_	A	archaeal Trm4	Stabilization of three-dimensional core	[9,209,210]
m^7^G_49_	A	Unknown		[36]
m^5^C_51_	A	Unknown	Stabilization of T-arm structure	[209]
m^5^C_52_	A	Unknown	Stabilization of T-arm structure	[209]
Ψ_54_ and Ψ_55_	A	Pus10	Stabilization of D-arm and T-arm interaction	[211,212,213,214]
m^1^Ψ_54_	A	Pus10 + TrmY	Stabilization of D-arm and T-arm interaction	[215,216,217]
m^5^U_54_ + m^5^s^2^U_54_	B/A	TrmFO + TtuA + TtuB + TtuC + TtuD + IscS/TrmA + TtuA? + TtuB? + α	Stabilization of D-arm and T-arm interaction (see main text) 2-Thiolation of m^5^s^2^U_54_ in tRNA is reviewed in Reference [239]	[10,11,21,22,23,24,67,97,98,134,218,219,220,221,222,223,224,225,226,227,228,229,230,231,232,233,234,235,236,237,238,239]
Um_54_	A	Unknown	Stabilization of D-arm and T-arm interaction	[44]
Ψ_55_	B/A	TruB/Pus10 or archaeal Cbf5 + α	Stabilization of D-arm and T-arm interaction In the case of *T. thermophilus*, Ψ_55_ is required for low-temperature adaptation (see main text) [248].	[10,11,18,19,20,23,25,36,44,64,67,211,212,213,214,240,241,242,243,244,245,246,247,248]
Cm_56_	A	Trm56	Stabilization of D-arm and T-arm interaction	[9,25,36,44,48,89,249,250,251]
m^2^G_57_	A	Unknown		[44,252]
m^1^I_57_	A	archaeal TrmI + unknown deaminase	Stabilization of T-arm structure	[44,253,254]
m^1^A_57_ and m^1^A_58_	A	archaeal TrmI	Stabilization of T-arm structure	[44,255,256,257,258]
m^1^A_58_	B	TrmI	Stabilization of T-arm structure	[11,23,67,204,259,260,261,262,263,264]

This table shows the nucleosides that are modified in tRNA from thermophiles. Most modifications are common to those in tRNA from mesophiles. Several modifications include derivatives and they are summarized as the derivatives (e.g., xm^5^U_34_ derivatives). In some cases, only modification enzymes from thermophiles have been reported. For example, although Q derivatives have not been confirmed in tRNA from *T. maritima*, the structure of QueA from *T. maritima* has been reported. In these cases, the modifications are listed here. The references for tRNA modifications and tRNA modification enzymes are mainly those for thermophiles. While there are many references for mesophiles, only representative references are cited. Where available, reviews of a modification and related proteins have been cited. Since modified nucleosides in tRNA from thermophilic eukaryotes have not been reported, modified nucleosides in eukaryotic tRNA have not been included here. The following modified nucleosides have been found in unfractionated tRNA from thermophiles. However, their positions and modified tRNA species are unknown: ac^6^A, hn^6^A, ms^2^hn^6^A, methyl-hn^6^A, m^2, 7^Gm, s^2^Um, and ac^4^Cm [56,97,98,99,100]. Abbreviations are as follows: A, archaea, B, eubacteria, and MT, methyltransferase. The “?” mark indicates the potential function speculated from the structure of the modified nucleosides.

**Table 2:** Thermophiles: their tRNA modifications and tRNA modification enzymes.

Species	Predicted Enzyme	Distinct tRNA Modifications and General Information	References
**Moderate Thermophiles**			
**Eubacteria**			
*Geobacillus stearothermophilus* (*Bacillus stearothermophilus*) 30–75 °C		Sequences of tRNA^Leu^ [18], tRNA^Phe^ [19], tRNA^Tyr^ [20], and tRNA^Val^2 [64] have been reported (Figure 2). The majority of modifications in tRNA are similar to those in *B. subtilis.* With increasing culture temperature, the extent of 2’-*O*-methylation in the tRNA mixture increases [309].	
	Gm_18_ (TrmH?)	Although *trmH* is not encoded in the *B. subtilis* genome, a *trmH*-like gene is encoded in the *G. stearothermophilus* genome. Gm_18_ has been found in tRNA^Tyr^ but not in tRNA^Leu^. This modification pattern suggests that the substrate tRNA specificity of *G. stearothermophilus* TrmH may be different from that of other known TrmH enzymes.	[20]
	D_17_, D_20_ and D_20a_ (Dus family protein?)	In *G. stearothermophilus* tRNA, D_17_, D_20_, and D_20a_ modifications have been reported. In *E. coli*, three Dus family proteins known as DusA, DusB, and DusC, produce D_20_ and D_20a_, D_17_, and D_16_, respectively [65,66]. In the *G. stearothermophilus* genome, however, only one gene is annotated as a *dus*-like gene. Therefore, D modifications in *G. stearothermophilus* cannot be explained by the tRNA substrate specificity of the known Dus proteins.	[17,19,64]
	m^1^A_22_ (TrmK?)	The m^1^A_22_ modification has been found in tRNA^Tyr^ and tRNA^Ser^ from *B. subtilis* and *Mycoplasma capricolum* [310,311]. *G. stearothermophilus* tRNA^Leu^ and tRNA^Tyr^ possess m^1^A_22_ [18,20]. The presence of a *trmK*-like gene in the genome of *G. stearothermophilus* has been reported [86].	[20,86]
	Gm_34_ (unknown MT)	*G. stearothermophilus* tRNA^Phe^ possesses Gm_34_ (Figure 2B) [19]. In contrast, the nucleoside at position 34 in *E. coli* tRNA^Phe^ is unmodified G. Given that *E. coli* TrmL acts only on tRNA^Leu^ isoacceptors [143], the 2′-*O*-methylation of G_34_ in tRNA^Phe^ from *G. stearothermophilus* is cannot be simply explained by the activity of known TrmL.	[19]
	m^6^A_37_ (YfiC; TrmG?)		[198]
**Archaea**			
*Methanobacterium thermoaggregans* Optimum growth temperature 60 °C		Sequences of tRNA^Asn^ and tRNA^Gly^ have been reported [8].	
*Methanobacterium thermoautotrophicum* 45–75 °C		The modified nucleosides in unfractionated tRNA are essentially common to those in tRNA from mesophilic methane archaea [97].	
*Methanococcus thermolithotrophicus* 17–62 °C		The modified nucleosides in unfractionated tRNA are essentially common to those in tRNA from mesophilic methane archaea [99].	
*Thermoplasma acidophilum* Optimum growth temperature 55–60 °C		Sequences of tRNA^Met^i [44,252], tRNA^Met^m [25], and tRNA^Leu^ [36] have been reported. Several recombinant tRNA modification enzymes have been used for biochemical studies.	
	s^4^U_8_ and s^4^U_9_ (ThiI? + α)	The s^4^U_9_ modification has been found in tRNA^Leu^ [36]. The sulfur donor for s^4^U formation is unknown [35].	[36]
	G^+^_13_ and G^+^_15_ (ArcTGT + ArcS?)	The G^+^_13_ modification has been found only in tRNA^Leu^ from *T. acidophilum* [36]. ArcTGT from *T. acidophilum* acts on both G13 and G15 in tRNA^Leu^ [47].	[36,47]
	m^2^_2_G_26_ (Trm1)		[89]
	ncm^5^U_34_ (Elp3?)		[36]
	m^1^G_37_ (Trm5)		[89]
	m^7^G_49_ (unknown MT)		[36]
	Cm_56_ (Trm56)	The presence of unusual *trm56*-like gene in the *T. acidophilum* genome has been reported in a bioinformatics study [250]. The Trm56 enzymatic activity has been confirmed via the recombinant protein [89]. *T. acidophilum* Trm56 exceptionally possesses a long C-terminal region in the SPOUT tRNA MT [312].	[89,250,312]
**Extreme**-**thermophiles and Hyper**-**thermophiles**			
**Eubacteria**			
*Aquifex aeolicus* Optimum growth temperature 85–94 °C		The partial sequence of tRNA^Cys^ has been reported [24] (Figure 2E). Several tRNA MT activities have been detected in the *A. aeolicus* cell extract using an *E. coli* tRNA mixture [24]. The tRNA modification enzymes listed below were characterized via recombinant proteins.	
	Gm_18_ (TrmH)		[74,77]
	D_20_ (Dus)	D_20_ exists in tRNA^Cys^. However, the nucleoside at position 20a is unmodified U [24]. Therefore, *A. aeolicus* Dus may act only on U_20_.	[24,82]
	m^2^G_26_, m^2^_2_G_26_, m^2^G_27_ and m^2^_2_G_27_ (Trm1)	*Aquifex aeolicus* exceptionally possesses Trm1 in eubacteria [24].	[24,95]
	I_34_ (TadA)		[104,105]
	mnm^5^U_34_ (MnmC2)	MnmC catalyzes the final methylation step of mnm^5^U synthesis. *Aquifex aeolicus* MnmC2 comprises only an MT domain.	[136]
	(MnmD; previously called GidA)		[140,141]
	k^2^C_34_ (TilS)		[111,112,113]
	m^1^G_37_ (TrmD)	The dimer structure of *A. aeolicus* TrmD is stabilized by inter-subunit disulfide bonds [165].	[160,162,165]
	m^7^G_46_ (TrmB)	TrmB proteins from thermophiles (*A. aeolicus* and *T. thermophilus*) possess a long C-terminal region.	[206,207,208]
	m^5^U_54_ and m^5^s^2^U_54_ (TrmFO)	The presence of *trmFO* gene in *A. aeolicus* genome was initially described in Reference [221].	[24,221]
	m^1^A_58_ (TrmI)		[257,262]
*Thermotoga maritima* 80–90 °C		Sequences of tRNA from *T. maritima* have not been reported. Recombinant proteins have been used for biochemical and structural studies.	
	hn^6^A (?)	hn^6^A was first identified in modified nucleosides from unfractionated tRNA from *T. maritima* [313]. Because hn^6^A was subsequently found in modified nucleosides from psychrophilic archaea [56], it is not a thermophile-specific modification. *Thermotoga maritima* and *Thermodesulfobacterium commune* exceptionally possess hn^6^A in eubacteria. The modification position in tRNA, modified tRNA species, and biosynthesis pathway of hn^6^A are unknown.	[56,313]
	s^4^U_8_ (ThiI + IscS)		[31,32]
	oQ_34_ (QueA)		[151]
	mnm^5^U_34_ (TrmE)		[138,139]
	t^6^A_37_ (TsaB, TsaC/TsaC2, TsaD and TsaE)		[190]
	ms^2^i^6^A_37_ (MiaB)		[194,195,196]
	m^1^G_37_ (TrmD)		[171]
	m^5^U_54_ and m^5^s^2^U_54_ (TrmFO and TtuA)	The m^5^s^2^U nucleoside has been found in unfractionated tRNA from *T. maritima* [97].	[97,134,221,222]
	Ψ_55_ (TruD)		[244,245,246,247]
	m^1^A_58_ (TrmI)		[257]
*Thermodesulfobacterium commune* Optimum growth temperature 70 °C	hn^6^A and ms^2^hn^6^A (?)	hn^6^A and ms^2^hn^6^A have been found in modified nucleosides from unfractionated tRNA from *T. commune*. The ms^2^hn^6^A modification may be derived from hn^6^A. So far, *T. commune* is the only eubacterium that possesses ms^2^hn^6^A in tRNA. The modification position in tRNA, modified tRNA species, and biogenesis pathway of hn^6^A and ms^2^hn^6^A are unknown.	[313]
*Thermus flavus* Optimum growth temperature 70 °C		Partial purification of tRNA m^1^A58 MT has been reported: the activity of tRNA m^7^G_46_ MT has also been described [204].	
*Thermus thermophiles* 50–83 °C		Sequences of tRNA^Met^f1 [21], tRNA^Met^f2 [21], tRNA^Ile^1 [67], tRNA^Asp^ [23], and tRNA^Phe^ [10,11] have been reported (Figure 2). Partial sequences of tRNA^Ser^_GGA_ [259], tRNA^Pro^_GGG_ [314], and tRNA^Pro^_GGA_ [314] have been determined. The modification extent of Gm_18_, m^5^s^2^U_54_ and m^1^A_58_ changes with the culture temperature. At high temperatures (>75 °C), m^7^G_46_ [11], m^5^s^2^U_54_ [230], and m^1^A_58_ [260] modifications are essential for survival. At low temperatures (<55 °C), Ψ_55_ is essential for survival [248] and m^5^U_54_ supports this effect [225] (see the main text). Recombinant proteins have been used for biochemical and structural studies.	
	m^2^G_6_ (TrmN)		[10,11,13,14,15]
	Gm_18_ (TrmH)		[10,11,21,23,30,69,70,71,72,73,75,76,78,79,80,81]
	D_20_ and D_20a_ (DusA)		[10,11,23,67,83,84,85]
	Cm_34_ and cmnm^5^Um_34_ (TrmL)		[144]
	Ψ_39_ and Ψ_40_ (TruA)		[10,11,18,23,202]
	m^7^G_46_ (TrmB)		[10,11,21,23]
	m^5^U_54_ and m^5^s^2^U_54_ (TrmFO + TtuA + TtuB + TtuC + TtuD + IscS)		[10,11,17,21,23,67,218,219,220,221,222,223,224,225,226,227,228,229,230,231,232,233,234,235,236,239]
	Ψ_55_ (TruB)		[10,11,21,23,67,248]
	m^1^A_58_ (TrmI)		[11,30,257,259,260,261,263,264]
**Archaea**			
*Aerophyrum pernix* 80–100 °C	Ψ_13_ and Ψ_15_ (archaeal Pus7 and H/ACA guide RNA system)	A guide RNA for Ψ formation has been predicted based on genome sequencing.	[46]
*Archaeoglobus fulgidus* 60–95 °C		Modified nucleosides in unfractionated tRNA from *A. fulgidus* have been reported [97].	
	agm^2^C_34_ (TiaS)		[116,118,119,315]
*Methanocaldcoccus igneus* (*Methanococcus igneus*; *Methanotorris igneus*) 45–91 °C		Modified nucleosides in unfractionated tRNA from *M. igneus* have been reported [56,99].	
*Methanocaldococcus infernus* 55–92 °C	cm^5^U_34_ (Elp3)		[142]
*Methanocaldcoccus jannashii* (*Methanococcus janaschii*) 48–94 °C		Although sequences of tRNA are unknown, the recombinant proteins listed below have been used for biochemical and structural studies.	
	m^2^G_6_ (Trm14)		[12]
	G^+^_15_ (ArcTGT + ArcS)		[51,59]
	Cm_34_ and Um_39_ (L7Ae, Nop5, aFib, Box C/D guide RNA system)		[316]
	m^1^G_37_ (Trm5)		[159,161,163,164,166,167,168,169,170]
	imG2_37_ (Trm5b + Taw1)		[173,179]
	yW-86_37_ (Taw2)		[174]
	m^5^C_48_ and m^5^C_49_ (archaeal Trm4)		[210]
	Ψ_54_ and Ψ_55_ (Pus10)		[211,212,213,214]
	m^1^Ψ_54_ (Pus10 + TrmY)		[215,216,217]
	Ψ_55_ (archaeal Cbf5)		[240]
*Methanopyrus kandleri* 84–110 °C (Strain 116: up to 122 °C)		Many unique modified nucleosides have been found in unfractionated tRNA [100]. tRNAs likely contain many 2’-*O*-methylated nucleosides derived from the C/D box guide RNA system [17].	
	ac^6^A (?)	The ac^6^A nucleoside has been purified from the modified nucleosides in unfractionated tRNA and its structure determined. The modification site, modified tRNA species, and biosynthesis pathway are unknown.	[100]
	U_8_ (CDAT8)		[16]
*Methanothermus fervidus* 80–97 °C		Only tRNA genes were reported in an early study [317].	
*Nanoarchaeum equitans* 70–98 °C		A unique tRNA processing system has been found [318,319]. The processing of small RNAs in *N. equitans* is reviewed in Reference [320].	
	m^1^G_37_ and imG2_37_ (Trm5a)		[176]
	m^5^U_54_ (TrmA-like protein)		[237]
*Pyrobaculum aerophilum* Optimum growth temperature 100 °C	Cm_56_ (L7Ae, Nop5, aFib, Box C/D guide RNA system)	Cm_56_ in tRNA is generally produced by Trm56. However, this modification in *P. aerophilum* is synthesized by the C/D box guide RNA system.	[249]
*Pyrobaculum calidifontis* 90–95 °C	G^+^_15_ (ArcTGT + QueF-like protein)	Eubacterial QueF catalyzes the conversion from preQ_0_ to preQ_1_. In *P. caldifontis*, however, QueF-like protein catalyzes the conversion from preQ_0_ at position 15 in tRNA to G^+^_15_.	[60,62,63]
*Pyrobaculum islandicum* Optimum growth temperature 100 °C		Modified nucleosides in unfractionated tRNA from *P. islandicum* have been reported [97].	
*Pyrococcus abyssi* Optimum growth temperature 96 °C		No tRNA sequence has been determined. However, the tRNA modification enzymes listed below have been characterized.	
	m^2^G_10_ and m^2^_2_G_10_ (archaeal Trm11, Trm-G10 enzyme, Trm-m22G10 enzyme)		[40,41]
	Ψ_13_ and Ψ_35_ (archaeal Pus7 and H/ACA guide RNA system)		[46]
	Cm_34_ and Um_39_ (L7Ae, Nop5, aFib, and C/D box guide RNA system)	Cm_34_ and Um_39_ in tRNA^Trp^ are formed by the C/D box guide RNA system in which the intron functions as a guide RNA.	[154,155]
	m^1^G_37_ (Trm5b)		[180]
	m^1^G_37_ and imG2_37_ (Trm5a)		[176,177,179]
	imG-14_37_ (Taw1)		[173,175]
	t^6^A37 (Kae1)		[185]
	(KEOPS complex)		[184]
	(Sua5 + KEOPS complex)		[187,189]
	m^5^C_48_ and m^5^C_49_ (archaeal Trm4 + archaese)		[209]
	m^5^U_54_ (TrmA-like protein, PAB0719)		[237,238]
	Ψ_55_ (Cbf5 + Nop10)		[241]
	Cm_56_ (Trm56)		[249]
	m^1^A_57_ and m^1^A_58_ (archaeal TrmI)		[255,256,257,258]
*Pyrococcus furiosus* Optimum growth temperature 100 °C		Modified nucleosides in unfractionated tRNA from *P. furiosus* have been reported [98]. Activity of several tRNA modification enzymes has been detected in the cell extract of *P. furious* [9].	
	m^2^G_6_ (Trm14)		[13,15]
	m^2^G_10_ and m^2^_2_G_10_ (archaeal Trm11, Trm-G10 enzyme, Trm-m22G10 enzyme)		[42]
	G^+^_15_ (ArcTGT)		[57]
	m^2^G_26_ and m^2^_2_G_26_ (Trm1)		[91,92]
	t^6^A37 (KEOPS complex)		[188]
	Ψ_54_ and Ψ_55_ (Pus10)		[212,214]
	Ψ_55_ (Cbf5 + Nop10 + Gar1)		[242]
*Pyrococcus horikoshii* 80–102 °C		The crystal structure of Nop5 in the C/D box guide RNA system from *P. horikoshii* has been solved [321].	
	G^+^_15_ (ArcTGT)		[50,52,53,54,55,89]
	m^2^G_26_ and m^2^_2_G_26_ (Trm1)		[89,93]
	yW-86_37_ (Taw2)		[174]
	m^5^s^2^U_54_ (TtuA)		[233]
	Cm_56_ (Trm56)		[251]
*Pyrodictium occultum* Optimum growth temperature 105 °C		Modified nucleosides in unfractionated tRNA have been analyzed and many 2’-*O*-methylated nucleosides found [97,98]. mimG was originally found among the modified nucleosides in tRNAs from *P. occultum*, *Sulfolobus solfaraticus*, and *Thermoproteus neutrophilus* [322]. Although the melting temperature of *P. occultum* tRNA^Met^i transcript is only 80 °C and that of native tRNA^Met^i is more than 100 °C (see main text) [323].	
*Pyrolobus fumarii* This archaeon can survive at 113 °C.		Modified nucleosides in unfractionated tRNA have been analyzed [324].	
*Stetteria hydrogenophila* Optimum growth temperature 95 °C		Modified nucleosides in unfractionated tRNA have been analyzed and methyl-hn^6^A, ms^2^hn^6^A, and m^2, 7^Gm identified [56].	
*Sulfolobus acidocaldarius* Optimum growth temperature 75–80 °C		Sequence of tRNA^Met^i has been reported [44]. The m^1^I_57_ modification was originally found in tRNAs from *S. acidocaldarius* and *Haloferax volcanii* [253]. G^+^ was first isolated from the nucleosides in *S. acidocaldarius* tRNAs and its structure determined [49]. The structures of wyosine derivatives (imG-14 and imG2) have been determined by using the nucleosides from *S. acidocaldarius* tRNAs [325].	
	m^1^A_9_ (archaeal Trm10)		[37,38]
	Ψ_13_ and Ψ_35_ (archaeal Pus7 and H/ACA guide RNA system)		[46]
	Cm_32_ (archaeal TrmJ)		[96]
*Sulfolobus solfaraticus* 55–90 °C		mimG was originally found among the modified nucleosides in tRNAs from *P. occultum*, *S. solfaraticus*, and *Thermoproteus neutrophilus* [322]. The structure of box C/D RNP from *S. solfaraticus* has been reported [326].	
	agm^2^C (TiaS)	The identification of agm^2^C_34_ in *Haloarcula marismortui* tRNA^Ile^2 and the presence of agm^2^C in *S. solfaraticus* tRNA have been reported.	[117]
	Ψ_13_ and Ψ_35_ (archaeal Pus7 and H/ACA guide RNA system)	Generally, Ψ_35_ in tRNA^Tyr^ is synthesized by archaeal Pus7. However, Pus7 from *S. solfaraticus* possesses weak Ψ_13_ formation activity but not Ψ_35_ formation activity. In *S. solfaraticus* and *A. pernix*, a guide RNA for Ψ_35_ formation exists.	[46]
	imG2_37_ (Trm5a; SSO2439 protein)	Trm5a (SSO2439 protein) does not possess m^1^G_37_ formation activity and is used only for imG2 formation.	[178]
	mimG_37_ (Taw3)		[180]
*Sulfolobus tokodaii* This archaeon can survive at 87 °C.	Ψ_13_ and Ψ_35_ (archaeal Pus7 and H/ACA guide RNA system)		[46]
	t^6^A_37_ (Sua5)		[327,328,329]
*Thermococuus celer* This archaeon can survive at 85 °C.		Although tRNA genes were analyzed in an early study [330], there is no information on tRNA modifications.	
*Thermococcus kodakarensis* (*Thermococcus kodakaraensis*; *Pyrococcus kodakarensis*) 65–100 °C	m^1^A_9_ and m^1^G_9_ (archaeal Trm10)		[37,39]
	m^2^G_10_ and m^2^_2_G_10_ (archaeal Trm11, Trm-G10 enzyme, Trm-m22G10 enzyme)		[43]
	G^+^_15_ (ArcTGT)		[47]
	m^5^U_54_ (TrmA-like protein)		[237]
*Thermoproteus neutrophilus* Optimum growth temperature 85 °C		Modified nucleosides in unfractionated tRNA have been analyzed [97]. mimG was originally found among the modified nucleosides in tRNAs from *P. occultum*, *S. solfaraticus*, and *T. neutrophilus* [322].	

Only distinct modifications that have been investigated are listed by thermophile species. In many cases, only tRNA modification enzymes (rather than modifications) have been studied by using recombinant proteins. For example, the presence of the m^7^G_46_ modification has not been confirmed in tRNA from *A. aeolicus*, but TrmB (tRNA m^7^G_46_ MT) has been characterized through the recombinant protein. In this case, m^7^G_46_ (TrmB) is listed in the section “*Aquifex aeolicus*”. The moderate thermophiles and extreme-thermophiles along with hyper-thermophiles are separated. Transfer RNA modifications in thermophilic eukaryotes are unknown. Abbreviation: MT, methyltransferase.

## Supplementary Materials

The following are available online at http://www.mdpi.com/2076-2607/6/4/110/s1, Table S1: Abbreviations of modified nucleosides, Table S2: Crystal structural studies on tRNA modification enzymes from thermophiles.
