# Supplementary material for: Transfer RNA Modification Enzymes from Thermophiles and Their Modified Nucleosides in tRNA
Source: Microorganisms. 2018 Oct 20;6(4):110. doi: 10.3390/microorganisms6040110 (PMC6313347; doi:10.3390/microorganisms6040110)
Supplement: Supplementary file 1 [file microorganisms-06-00110-s001.pdf]

Supplementary Table S1 Abbreviations of modified nucleosides.

Abbreviation	Modified nucleosides
s^4U	4-thiouridine
D	dihydrouridine
m^1A	N^1-methyladenosine
Cm	2′-*O*-methylcytidine
ms^2i^6A	2-methylthio-N^6-isopentenyladenosine
Ψ	pseudouridine
m^5U	5-methyluridine
Gm	2′-*O*-methylguanosine
m^7G	7-methylguanosine
Q	queuosine
m^6A	N^6-methyladenosine
m^2G	N^2-methylguanosine
$m^2{}_2G$	N^2,N^2-dimethylguanosine
i^6A	N^6-isopentenyladenosine
m^5s^2U	5-methyl-2-thiouridine
G^+	archaeosine
t^6A	N^6-threonylcarbamoyladenosine
ncm^5U	5-carbamoylmethyluridine
m^1G	1-methylguanosine
m^5C	5-methylcytidine
Um	2′-*O*-methyluridine
m^1I	1-methylinosine
s^2C	2-thiocytidine
m^5Cm	5, 2′-*O*-dimethylcytidine
mimG	methylwyosine
m^1Im	1, 2′-*O*-dimethylinosine
$m^2{}_2Gm$	N^2, N^2, 2′-*O*-trimethylguanosine
m^2, ^7Gm	N^2, 7, 2′-*O*-trimethylguanosine

Am	2′-*O*-methyladenosine
I	inosine
k^2C	lysidine
agm^2C	agmatidine
mnm^5U	5-methylaminomethyluridine
cm^5U	5-carboxymethyluridine
cnm^5U	5-cyanomethyluridine
$cmnm^5Um$	5-carboxymetylaminomethyl-2′-*O*-methyluridine
$m^1\Psi$	1-methylpseudouridine
ac^6A	N^6-acetyladenosine
hn^6A	N^6-hydroxynorvalylcarbamoyladenosine
ms^2hn^6A	2-methylthiio- N^6-hydroxynorvalylcarbamoyladenosine
methyl-hn^6A	methylated hn^6A*
s^2Um	2-thio-2′-*O*-methyluridine
ac^4Cm	4-acetyl-2′-*O*-methylcytidine
oQ	epoxyqueuosine
imG2	isowyosine
yW-86	7-aminocarboxypropyldemethylwyosine
imG-14	4-demethylwyosine

* Precise structure is unknown.

Supplementary Table S2 Crystal structural studies on tRNA modification enzymes from thermophiles.

**tRNA modification enzyme(s)**	**Source (thermophile species)**	**PDB ID**	**Reference(s)**
Archaeal Trm14	*Pyrococcus furiosus*	3TLJ, 3TM4, 3TM5	[15]
TrmN	*Thermus thermophilus*	3TMA	[15]
CDAT8	*Methanopyrus kandleri*	3G8Q	[16]
ThiI	*Thermotoga maritima*	4KR6, 4KR7, 4KR9	[32]
Archaeal Trm10	*Sulfolobus acidocaldarius*	5A7Z, 5A7Y, 5A7T	[38]
	Thermococcus kodakarensis	6EMS, 6EMT, 6EMU, 6EMV	[39]
Archaeal Trm11	*Thermococcus kodakarensis*	5E71, 5E72	[43]
ArcTGT	*Pyrococcus horikoshii*	1IT7, 1IT8, 1IQ8	[54,55]
TrmH	*Thermus thermophilus*	1V2X	[75]
	Aquifex aeolicus	1ZJR	[77]
DusA	*Thermus thermophilus*	3B0V, 3B0U, 3B0P	[84]
Trm1	*Pyrococcus horikoshii*	2DUL, 2DYY, 2EJT, 2EJU, 2YTZ	[93]
	Aquifex aeolicus	3AXS, 3AXT	[95]
Archaeal TrmJ	*Sulfolobus acidocaldarius*	4CNF, 4CNG	[96]
TadA	*Aquifex aeolicus*	1WWR	[104,105]
TilS	*Aquifex aeolicus*	1WY5, 2E21, 2E89	[112,113]
TiaS	*Archaeoglobus furgidus*	3AU7, 3AMU, 3AMT	[119]
MnmC2	*Aquifex aeolicus*	3VYW	[136]
MnmE	*Thermotoga maritima*	1XZP, 1XZQ	[138]
MnmG	*Aquifex aeolicus*	2ZXI, 2ZXH	[141]
TrmL	*Thermus thermophilus*	5CO4	[144]
QueA	*Thermotoga maritima*	1VKY	[151]
TrmD	*Aquifex aeolicus*	1OY5	[160]
Archaeal Trm5	*Methanocaldococcus janaschii*	3AY0, 2YX1, 2ZZN	[166–168]
Trm5a	*Pyrococcus abyssi*	5WT1, 5WT3	[177]
Trm5b	*Pyrococcus abyssi*	5YAC	[180]
Taw1	*Pyrococcus horikoshii*	2YX0	[172]
	Methanocaldococcus janaschii	2Z2U	[173]
Taw2	*Pyrococcus horikoshii*	3A25, 3A26	[174]
	Methanocaldococcus janaschii	3A27	[174]
Taw3	*Sulfolobus solfataricus*	1TLJ	[178]
KEOPS complex	*Methanocaldococcus janaschii* and	3ENQ, 3ENC, 3ENH, 3ENO, 2VWB	[184,185]

	Pyrococcus furiosus		
Sua5	*Pyrococcus abyssi*	6F89, 6F87, 6F8Y	[189]
	Sulfolobus tokodaii	2EQA, 2YV4, 3AJE, 4E1B	[327,328]
TruA	*Thermus thermophilus*	1VS3	[202]
Archaeal Trm4	*Methanocaldococcus janaschii*	3A4T, 3AJD	[210]
TrmY	*Methanocaldococcus janaschii*	3AI9, 3AIA	[215]
TrmFO	*Thermus thermophilus*	3G5Q, 3G5R, 3G5S	[223]
TtuA	*Thermus thermophilus*	5B4F, 5B4E	[236]
	Thermotoga maritima	5MKQ, 5MKO	[134]
	Pyrococcus horikoshii	3VRH	[233]
TtuA and TtuB complex	*Thermus thermophilus*	5GHA	[236]
Archaeal TrmA	*Pyrococcus abyssi*	2JJQ, 2VS1	[238]
TruB	*Thermotoga maritima*	1R3E, 1R3F, 1ZE1, 1ZE2, 2AB4	[244,246,247]
Trm56	*Pyrococcus horikoshii*	2YY8	[251]
Archaeal TrmI	*Pyrococcus abyssi*	3LGA, 3LHD, 3MB5	[256]
TrmI	*Thermus thermophilus*	5C0O, 5C1I, 2PWY	[261,264]
	Aquifex aeolicus	2YVL	[262]
Box C/D RNP	*Sulfolobus solfataricus*	5GIN, 5GIO, 5GIP	[326]

In this table, only the published crystal structures from thermophiles are listed. Numerous unpublished protein structures, which seem to be related to tRNA modifications, are deposited in Protein Data Bank. In this table, the structures of tRNA modification enzymes from mesophiles are not cited.
